# *Proteus mirabilis* Urease: Unsuspected Non-Enzymatic Properties Relevant to Pathogenicity

**DOI:** 10.3390/ijms22137205

**Published:** 2021-07-04

**Authors:** Matheus V. C. Grahl, Augusto F. Uberti, Valquiria Broll, Paula Bacaicoa-Caruso, Evelin F. Meirelles, Celia R. Carlini

**Affiliations:** 1 Laboratory of Neurotoxins, Brain Institute of Rio Grande do Sul (BRAINS) and Graduate Program in Medicine and Health Sciences, Pontifícia Universidade Católica do Rio Grande do Sul (PUCRS), Porto Alegre CEP 90610-000, RS, Brazil; matheusgrahl@hotmail.com (M.V.C.G.); afuberti@gmail.com (A.F.U.); 2 Graduate Program in Cellular and Molecular Biology, Center of Biotechnology, Universidade Federal do Rio Grande do Sul (UFRGS), Porto Alegre CEP 91501-970, RS, Brazil; valbroll@gmail.com; 3 Laboratory of Neurotoxins, Brain Institute of Rio Grande do Sul (BRAINS) and Graduate Program in Cellular and Molecular Biology, Pontifícia Universidade Católica do Rio Grande do Sul (PUCRS), Porto Alegre CEP 90610-000, RS, Brazil; paula.caruso@edu.pucrs.br; 4 Laboratory of Neurotoxins, Brain Institute of Rio Grande do Sul (BRAINS), Pontifícia Universidade Católica do Rio Grande do Sul (PUCRS), Porto Alegre CEP 90610-000, RS, Brazil; evelin.meirelles@acad.pucrs.br; 5 Laboratory of Neurotoxins, Brain Institute of Rio Grande do Sul (BRAINS) and School of Medicine, Pontifícia Universidade Católica do Rio Grande do Sul (PUCRS), Porto Alegre CEP 90610-000, RS, Brazil

**Keywords:** *Proteus mirabilis*, urease, virulence factors, pathogenesis

## Abstract

Infection by *Proteus mirabilis* causes urinary stones and catheter incrustation due to ammonia formed by urease (PMU), one of its virulence factors. Non-enzymatic properties, such as pro-inflammatory and neurotoxic activities, were previously reported for distinct ureases, including that of the gastric pathogen *Helicobacter pylori*. Here, PMU was assayed on isolated cells to evaluate its non-enzymatic properties. Purified PMU (nanomolar range) was tested in human (platelets, HEK293 and SH-SY5Y) cells, and in murine microglia (BV-2). PMU promoted platelet aggregation. It did not affect cellular viability and no ammonia was detected in the cultures’ supernatants. PMU-treated HEK293 cells acquired a pro-inflammatory phenotype, producing reactive oxygen species (ROS) and cytokines IL-1β and TNF-α. SH-SY5Y cells stimulated with PMU showed high levels of intracellular Ca^2+^ and ROS production, but unlike BV-2 cells, SH-SY5Y did not synthesize TNF-α and IL-1β. Texas Red-labeled PMU was found in the cytoplasm and in the nucleus of all cell types. Bioinformatic analysis revealed two bipartite nuclear localization sequences in PMU. We have shown that PMU, besides urinary stone formation, can potentially contribute in other ways to pathogenesis. Our data suggest that PMU triggers pro-inflammatory effects and may affect cells beyond the renal system, indicating a possible role in extra-urinary diseases.

## 1. Introduction

*Proteus mirabilis*, a rod-shaped Gram-negative bacterium of the gut microbiome, is an opportunistic uropathogen. Formation of bladder and kidney stones are characteristic of *P. mirabilis*-induced urinary tract which can result in permanent renal damage [1]. Estimates are that half of the patients catheterized for up to one week develop a catheter-associated urinary infection, 44% of which are due to *P. mirabilis* infection [2]. *Proteus mirabilis* produces a urea-inducible urease (PMU) that acts as a virulence factor [1,3]. Pathogenesis is sustained by other virulence factors that work cooperatively with PMU, such as fimbriae and adhesins [1,4]. PMU enables bacterial survival by hydrolyzing urea into carbon dioxide and ammonia, thus, providing nitrogen for the pathogen. The generated ammonia is toxic to the host cells and alkalinizes the urine, leading to precipitation of urinary salts and formation of stones, which protect the entrapped bacteria, clog the urinary tract, and arrest the urine flow [5,6]. *P. mirabilis* interacts with neutrophils and macrophages thereby eliciting a strong innate immune response [7]. The DNA-damaging monochloramine is formed from ammonia produced by urease and hypochlorous acid released by activated immune cells [8]. The NLRP3 inflammasome participates in pathogenesis induced by *P. mirabilis* resulting in interleukin-1β (IL-1β) production, thereby potentiating ammonia-induced tissue damage [9].

*Proteus* spp. can also cause osteomyelitis, mastoiditis, wound infections, otitis, neonatal meningitis, hemorrhagic meningoencephalitis, adult post-neurosurgical meningitis [10,11,12,13] and hepatic encephalopathy in patients with acute liver failure [14]. Moreover, *P. mirabilis* has been recently implicated in the pathogenesis of Parkinson’s disease [15,16]. A previous urinary infection by *P. mirabilis* predisposes, decades later, to the development of rheumatoid arthritis. This predisposition was attributed to a molecular mimicry between cartilage α-2 (XI) collagen and the bacterial urease [13,17].

There are no current vaccines for *P. mirabilis*, and multidrug-resistant isolates are on rise [18]. The relevance of PMU to formation of urinary stones and catheter encrustation has long been acknowledged [19], but despite intense efforts no inhibitor of the enzyme’s activity has reached the market yet [20]. The involvement of PMU in other *P. mirabilis*-associated pathologies has not been characterized. As the most prominent virulence factor of *P. mirabilis*, there is an urge for a better understanding of the contributions of PMU not only to urinary infections, but also to the extra-urinary pathologies associated with this bacterium.

Ureases from different sources share at least 55% identity of their amino acid sequences although differing in their quaternary structures [21]. *P. mirabilis* urease has three subunits arranged as (αβγ)_3_ oligomer. The enzyme’s active site is located on PmUreα, but this subunit is devoid of enzymatic activity when isolated [22]. Ureases are moonlighting proteins which display many other unrelated biological properties besides the urea hydrolyzing activity (reviewed in [23,24]). The ammonia-independent toxicity of ureases was initially described for canatoxin, an isoform of urease of the legume jack bean (*Canavalia ensiformis*), which is lethal to mice and rats, causing seizures that precede the animal’s death [25]. Moreover, canatoxin promotes exocytosis in several types of mammalian cells and displays pro-inflammatory and neurotoxic activities [25,26,27,28]. Consistent with a role in plant defense, insecticidal [29] and fungitoxic [30] properties were described for canatoxin. Later, we reported that ureases of other plants (soybean, cotton) and the bacterial enzymes of *Helicobacter pylori* [31,32,33,34,35] and *Sporosarcina* (*Bacillus*) *pasteurii* [36] also present most of the non-enzymatic activities described for canatoxin (reviewed in [37]). In the case of *H. pylori* urease (HPU), the recombinant purified protein (nanomolar concentrations) was shown to activate platelets promoting aggregation [31] and conversion into a pro-inflammatory phenotype [32]. Purified HPU activates neutrophils, both in vitro and in vivo, inhibiting their apoptosis and inducing production of reactive oxygen species [34]. HPU promoted the release of pro-inflammatory cytokines from human endothelial and microvasculature cells and increased their paracellular permeability by destabilizing cell–cell junctions [33]. Moreover, it showed angiogenic activity upon endothelial and gastric epithelial cells [33,35]. Altogether, these biological properties suggest that HPU may potentially contribute to inflammation leading to gastritis and carcinogenesis associated with the chronic infection by *H. pylori*.

In the light of these facts, here we aimed to determine if PMU is also a multifunctional protein that could potentially contribute in other ways to the pathogenicity of *Proteus* spp. By carrying out several bioassays with a purified recombinant PMU on human and murine cells, we were able to demonstrate its pro-inflammatory and neurotoxic properties. We also showed the sub-cellular localization of PMU in the cells’ nucleus and cytoplasm, consistent with the presence of nuclear localization sequences in the molecule.

## 2. Results

### 2.1. Moonlighting (Non-Enzymatic) Properties of PMU 

#### 2.1.1. Aggregation of Human Platelets by PMU and Its Subunits

**Figure 1:**
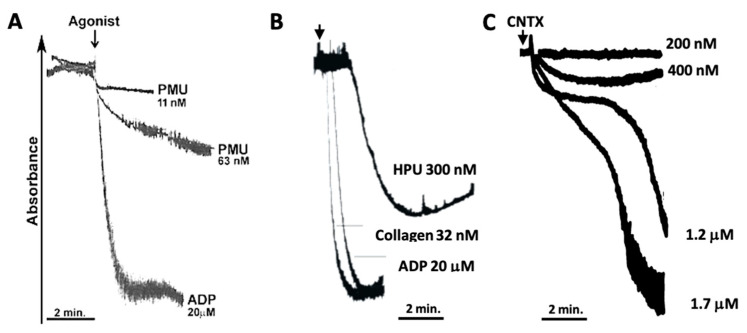
Aggregation of platelets induced by *P. mirabilis* urease and other ureases. Turbidimetric assay of platelet aggregation induced by ureases. The reaction started by addition of the agonist (urease, ADP, or collagen) to a platelet-rich plasma suspension and the aggregation response (a decrease in absorbance at 630 nm) was monitored for 5 min. The tracings were superimposed to facilitate comparison. Panel (**A**) aggregation of human platelets in the presence of PMU (11 and 63 nM) or 20 µM ADP. Typical results. Panel (**B**) aggregation of rabbit platelets in the presence of *H. pylori* urease (HPU, 300 nM), collagen (32 nM) or ADP (20 mM). Data adapted from [31]. Panel (**C**) aggregation of human platelets in the presence of canatoxin (CNTX, 200 nM, 400 nM, 1.2 mM, and 1.7 mM). Data adapted from [39].

The ability to activate blood platelets, coupled with exocytosis of their dense granules, is one of the properties shared by plant and bacterial ureases that contributes to their pro-inflammatory effects [25,26,31,36,38]. Moreover, we demonstrated that human platelets acquired a pro-inflammatory phenotype when stimulated by *H. pylori* urease [32]. Here we showed that PMU (17 µg.mL^−1^, 63 nM) is also able to induce aggregation of human platelets (Figure 1). PMU-induced aggregation had a slower rate compared to the platelets’ response to ADP (20 µM, supramaximal dose), although the ~300-fold lower dose of the protein eventually yielded a similar extent of aggregation.

Figure 1 illustrates the tracings of platelet aggregation as induced by the urease of *H. pylori* urease (HPU) and by canatoxin, a plant urease. PMU is apparently more active than the two other ureases. Platelet aggregation is consequent to the urease’s eicosanoid-mediated exocytosis-inducing effect that leads to the release of ADP from the platelet’s dense granules, which is the final aggregation inducer [26,31,36,38,39].

#### 2.1.2. Effects of PMU in Cell Cultures

Aiming to investigate if PMU displays other biological effects, besides platelet-aggregating activity, that could be relevant in the context of pathogenesis by *Proteus mirabilis*, a series of bioassays were conducted employing three lines of cultured cells. HEK293 cells (derived from a human embryonic kidney) were chosen considering the putative role of PMU in the inflammatory reactions associated with urinary and kidney infections caused by *P. mirabilis*. To investigate a possible neurotoxic property of PMU, additional studies were performed on human neuroblastoma SH-SY5Y and murine microglial BV-2 cells. 

**Figure 2:**
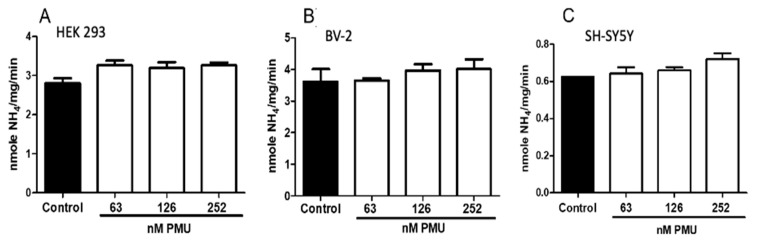
Accumulation of ammonia in the medium of cells cultured in the presence of PMU. Cultured cells (**A**) kidney, HEK293, (**B**) murine microglia, BV-2, and (**C**) human neuroblastoma, SH-SY5Y, were incubated at 37 °C for 24 h with 20 mM sodium phosphate buffer pH 7.0 (NaPB 7.0) (control), or PMU at 63, 126 or 252 nM concentrations. The levels of ammonia accumulated in the cultures’ supernatants were monitored by a colorimetric assay. The results are expressed in nanomoles NH_4_/mg cell protein/min. Results are expressed as mean ± SEM (*n* = 9–12 for the control and 9–14 for treatments). The data were analyzed by one-way parametric ANOVA with a Dunnett post-test.

After 24 h of incubation with all tested doses of PMU, the ammonia concentration in the media of the three types of cell cultures remained below the physiological levels of ~25 μM (Figure 2) [40], indicating a non-significant contribution of PMU’s enzyme activity to any observed effects. This result suggests that inhibitors of urea hydrolysis alone would probably not counteract the pro-inflammatory activity of PMU as described here.

#### 2.1.3. Pro-Inflammatory Properties of PMU in HEK293 Cells

**Figure 3:**
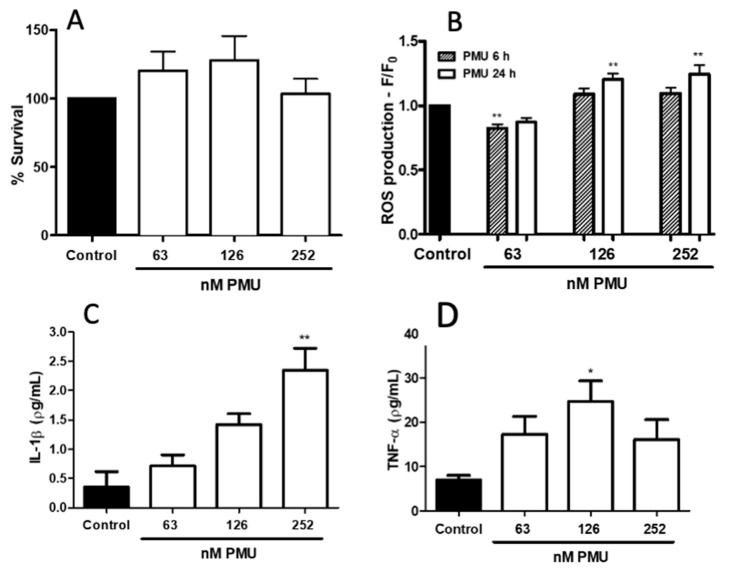
Effects of *Proteus mirabilis* urease on HEK293 cells. Cultured human kidney embryonic (HEK293) cells were incubated with NaPB 7.0 (control), or PMU at 63, 126 and 252 nM for 6 h and 24 h. In panel (**A**), cell culture viability was analyzed by the MTT test 24 h after exposure to a buffer or PMU. After the treatments, cultures’ supernatants were removed and cells were treated with MTT (5 mg/mL) for 4 h at 37 °C, then suspended in 100 μL DMSO. Absorbances were read at 570 nm. Mean ± SEM (*n* = 4–5 for the control and 5–8 for treatments). * *p* < 0.05, ** *p* < 0.01 vs. control. In panel (**B**), intracellular production of reactive oxygen species was assessed by pre-incubating the cells with CM-DFFDA (2 mM, 30 min) before exposure to a buffer or PMU, followed by fluorescence analysis (excitation 495 nm, emission 527 nm). Mean ± SEM (*n* = 10, controls; *n* = 8–10 for treatments) * *p* < 0.05, ** *p* < 0.01, vs. control. In panels (**C**,**D**), HEK293 cells were incubated for 6 h with the NaPB 7.0 (control), 63, 126, 252 nM PMU. Afterwards the cultures’ supernatants were collected for detection of IL-1β (panel A) and TNF-α (panel B) by ELISA. The data were analyzed by one-way parametric ANOVA with a Dunnett post-test, the results are mean ± SEM (*n* = 4–5, control, and 4–7 for each PMU dose). * *p* < 0.05, ** *p* < 0.01 vs. control.

As shown in Figure 3, PMU was not cytotoxic to HEK293 cells in the MTT assay, which reflects mitochondrial activity, after 24 h of incubation. 

Indicating a conversion into a pro-inflammatory phenotype, HEK293 cells produced ROS and secreted TNF-α and interleukin-1β after exposure to PMU for 6 h (Figure 3B–D). 

#### 2.1.4. Pro-Inflammatory Potential of PMU in CNS-Derived Cells

**Figure 4:**
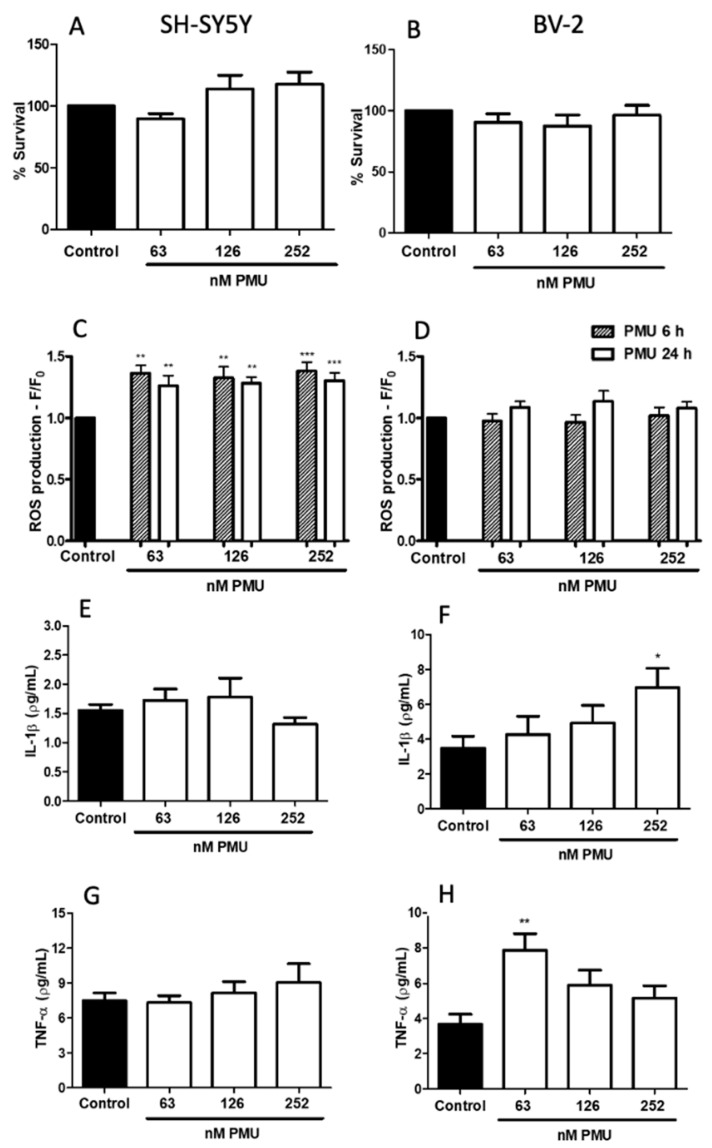
Neurotoxic and neuroinflammatory effects of *Proteus mirabilis* urease. Cultured neuroblastoma, SH-SY5Y (**A**,**C**,**E**,**G**) or microglial, BV-2 (**B**,**D**,**F**,**H**) cells were incubated with NaPB 7.0 (control), or 63, 126 and 252 nM PMU for 6 h (hatched columns) and 24 h (open columns). In panels **A**,**B**, cell culture viability was analyzed 24 h after exposure to a buffer or PMU. After the treatments, cultures’ supernatants were removed and the cell pellets were treated with 100 μL MTT (5 mg/mL) for 4 h at 37 °C, then suspended in 100 μL DMSO. Absorbances were read at 570 nm. Mean ± SEM (*n* = 4–5 for the control and 5–8 for treatments). * *p* < 0.05, ** *p* < 0.01 and *** *p* < 0.001 vs. control. In panels **C**,**D**, intracellular production of reactive oxygen species was assessed by pre-incubation of the cells with fluorophore CM-DFFDA (2 mM, 30 min) before exposure to a buffer or PMU, followed by fluorescence analysis (excitation 495 nm, emission 527 nm). Mean ± SEM (*n*= 5–10, controls; *n*= 5–16 for treatments) * *p* < 0.05, ** *p* < 0.01, *** *p* < 0.001 vs. control. In panels **E**–**F**, after incubation for 6 h with NaPB 7.0 (control) or PMU (63, 126, 252 nM) of SH-SY5Y (**E**,**G**), or BV-2 (**F**,**H**) cell cultures, the supernatants were collected for detection of IL-1β (**E**,**F** panels) and TNF-α (**G**,**H** panels) by ELISA. The data were analyzed by one-way parametric ANOVA with a Dunnett post-test, the results are the mean ± SEM (*n* = 4–6 for the control and 3–6 for treatments). * *p* < 0.05 and ** *p* < 0.001 vs. control.

**Figure 5:**
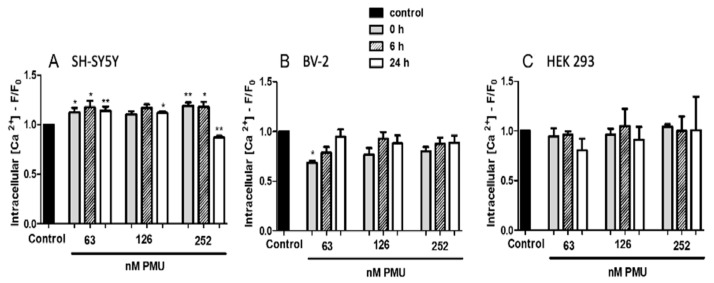
Levels of intracellular Ca^2+^ of cells cultured in the presence of PMU. Cultured (**A**) neuroblastoma, SH-SY5Y, (**B**) microglia, BV-2, and (**C**) kidney, HEK293, were incubated with the fluorophore calcium probe Fluo4 (0.33 mg/100 µL for 45 min), washed to remove the excess of the probe and then treated with PMU (63, 126, 252 nM) for 0, 6 or 24 h. Cells treated with NaPB 7.0 were taken as controls. The fluorescence (excitation 488 nm/emission 530 nm) intensity was then measured, and the values found for controls were considered as 1.0. The results are the mean ± SEM (*n* = 4 for the control and 4–5 for treatments). The data were analyzed by one-way parametric ANOVA with a Dunnett post-test. * *p* < 0.05 and ** *p* < 0.01 vs. control.

The neurotoxic potential of PMU was evaluated in cultured human neuroblastoma and murine microglial cells (Figure 4 and Figure 5). The MTT assay showed that, such as HEK293 cells, during the 24 h period of incubations with 63, 126, and 252 nM PMU, cell viability was not altered in both cell lines (Figure 4A,B).

Higher intracellular levels of ROS were detected in PMU-treated neuroblastoma cells in all doses and time points, whereas microglial cells did not produce ROS when exposed to PMU under the same conditions (Figure 4C,D).

A potential pro-inflammatory effect of PMU on the nervous system cells was assessed measuring the levels of the cytokines TNF-α and IL-1β in the supernatant of PMU-treated cultures of neuroblastoma or microglial cells. In the tested conditions, no production of IL-1β or TNF-α was detected in PMU-stimulated SH-SY5Y cells. On the other hand, BV-2 cells secreted both pro-inflammatory cytokines, indicating a neurotoxic and neuroinflammatory effect of PMU on microglial cells (Figure 4F,H).

#### 2.1.5. Modulation by PMU of Intracellular Calcium Levels

Consistent with its excitable properties, neuroblastoma cells had a dose- and time-dependent increase in intracellular Ca^2+^ levels in the presence of PMU, in almost all the doses and incubation times tested. On the other hand, the calcium content of microglial cells, as well as that of HEK293, remained constant upon incubation with PMU under similar conditions (Figure 5).

### 2.2. Internalization and Nuclear Localization of PMU 

**Figure 6:**
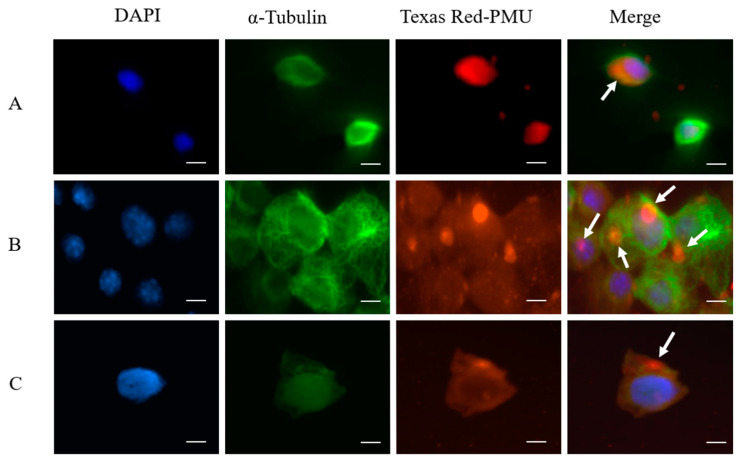
Fluorescence microscopy analysis of PMU-treated cells and nuclear localization sequences. Texas Red-labeled PMU (63 nM) was incubated for 1 h at room temperature with the cell lines (**A**) human neuroblastoma, SH-SY5Y, (**B**) murine microglia, BV-2, and (**C**) human embryonic kidney, HEK293. The cells were then treated with anti-α-Tubulin antibodies (1:1000, in green) to stain the cytoskeleton and with DAPI to label the nucleus (in blue). Cells were visualized under a fluorescence microscope. Scale-bars: 10 μm. The arrows in the merge view indicate the presence of PMU interacting with the cells in the cytoplasm, perinuclear region and in the nuclei. The pictures are representative of at least three independent assays.

The three cell types internalized Texas Red-labeled PMU after 1 h of incubation. Fluorescence microscopy showed the labeled protein as a punctate pattern in the cytoplasm, in the perinuclear region, or in the nucleus (Figure 6A–C), suggesting interactions with the cytoskeleton and/or nucleus.

**Figure 7:**
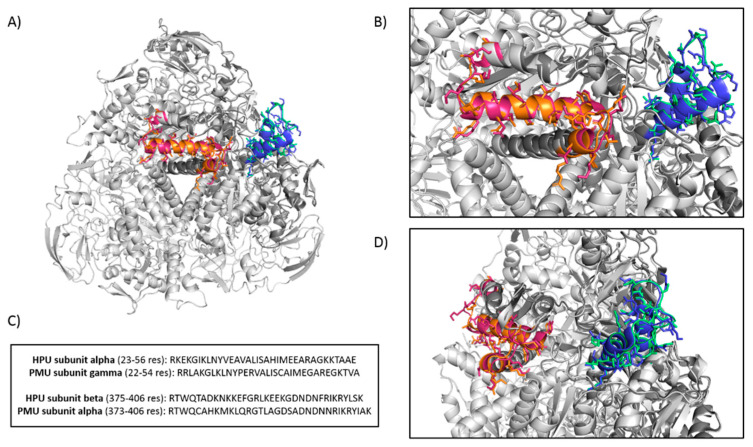
Nuclear localization sequences in *P. mirabilis* (PMU) and *H. pylori* (HPU) ureases. Panels (**A**–**D**) show the nuclear localization sequences (NSL) in *P. mirabilis* and *H. pylori* ureases. Panel (**A**) illustrates the 3D cartoon models of *P. mirabilis* urease in its (γβα)_3_ oligomer (light gray) superimposed to *H. pylori* urease in its (αβ)_3_ form (dark gray). The NLSs in PMU are pictured in orange (positions 22–54) and green (positions 575–608) and in HPU, they are shown in pink (positions 21–56) and blue (positions 613–646). The sequences corresponding to the NLS are presented in panel (**C**). Closer views of the two bipartite NLSs present in HPU and PMU are seen in Panels (**B**,**D**) (90 degree turn). Images were built with PyMOL.

The amino acid sequences of PMU and of HPU were analyzed by the cNLS Mapper software, which revealed two bipartite NLS sequences in both ureases, one located in the γ domain (PMU subunit γ or A; HPU subunit A) and the second tag located in their α domain (PMU subunit α or C; HPU subunit B). These analyses are shown in Figure 7 and Supplementary Figure S3. The HPU’s sequence _21_KKRKEK_26_, previously identified as a monopartite NSL [41], is part of a larger bipartite NLS found in the N-terminal half of its subunit A. According to the cNLS Mapper, scores in the range of 3.0 to 5.0 tag the protein to both the cytoplasm and the nucleus, while higher values address a preferential nuclear localization [42]. The scores determined by the cNLS Mapper for the ureases’ NLS varied from 4.4 to 5.6, corroborating the fluorescence microscopy results that displayed the labeled PMU in the corresponding subcellular compartments in the three cell lines (Figure 6). 

## 3. Materials and Methods

### 3.1. Plasmid Construction and Bacterial Strain

*Escherichia coli* HB101 carrying a pMID 1010 plasmid was a kind gift from Dr. Harry T. Mobley (University of Michigan Medical School, Ann Arbor, MI, USA). This plasmid contains the complete operon for PMU formed by eight genes in tandem *ure*R-*ure*D-*ure*ABC-*ure*EFG: three structural genes (*ure*A, *ure*B and *ure*C) and five genes encoding accessory and regulatory proteins (*ure*D, *ure*E, *ure*F, *ure*G and *ure*R), thus, encoding a fully active urease. Using Pfu DNA polymerase (Promega, Madison, WI, USA) and the primers PmUreD-5′ (AGGAGATATA**CCATGG**ATGCCTGACTTTTCTGAGAA) and PmUreBCureEFG-3′ (GTTAGCAGCC**GGATCC**TTAACGTCTCAACATACCTTT), the operon was amplified from *ure*D to *ure*G to yield a D*ureR* holoPMU operon (Supplementary Figure S1). The amplified DNA was cloned into a pGEM-T plasmid and then inserted into pET15b between NcoI and BamHI restriction sites. For protein expression, *E. coli* BL21(DE3)pLysS (Novagen, Germany) cells were transformed with the Δ*ureR* holo PMU::pET15b plasmid by heat shock (30 min ice bath, 45 s at 42 °C, 2 min ice bath).

### 3.2. Bacterial Growth and Induction of Proteus Mirabilis Urease

*E. coli* was cultured in lysogeny broth (LB), with 100 µg.mL^−1^ ampicillin and 40 µg.mL^−1^ chloramphenicol (Sigma-Aldrich, St. Louis, MO, USA). When the culture reached an OD_600_ of 0.7, protein expression was induced with 0.75 mM isopropyl-thiogalactoside (IPTG) (Fisher Scientific, Geel, Belgium) and 1 mM NiCl_2_, at 27 °C, 180 rpm, overnight, with addition of 100 µg.mL^−1^ ampicillin. Recombinant colonies expressing PMU were screened in urea segregation agar [43].

### 3.3. Crude Extract and Purification of PMU

The culture was centrifuged at 5800 *g* for 30 min at 4 °C. The pellet was resuspended in 20 mM sodium phosphate pH 7.0 (NaPB 7.0), centrifuged again, cells were suspended in the same buffer and disrupted using a Unique Ultrasonic Homogenizer (Hielscher Ultrasonics, Teltow, Germany), 20 pulses of 60 s, with a pause of 60 s between the pulses, in an ice bath. The lysate was centrifuged at 15,000× *g* for 40 min at 4 °C, the supernatant was dialyzed against NaPB 7.0 and then filtered on a 0.22 µm filter.

After dialysis, the crude extract was submitted to four sequential chromatographic steps in an ÄKTA apparatus (GE Healthcare, Little Chalfont, UK). The extract was applied into a Q-Sepharose^TM^ High Performance column (GE Healthcare, Little Chalfont, UK), equilibrated in NaPB 7.0, and washed with the same buffer to remove unbound proteins. The fraction with ureolytic activity was eluted between 0.3 and 0.56 M KCl in NaPB 7.0, in a 70 mL gradient, with a 2 mL.min^−1^ flow rate. The urease-enriched fractions were pooled and dialyzed against NaPB adjusted to pH 7.5 (NaPB 7.5), filtered on a 0.22 µm filter and then loaded into a Source^TM^ 15Q column (GE Healthcare, Little Chalfont, UK), equilibrated in NaPB 7.5. Elution was performed with a 50 mL linear gradient of KCl in NaPB 7.5, with a 1 mL.min^−1^ flow rate. The active fractions, eluted between 0.24 and 0.48 M KCl, were pooled and concentrated using a Vivaspin^TM^ (GE Healthcare, Little Chalfont, UK) device with a 100 kDa cut-off. This material was then submitted to size exclusion chromatography on a Superdex 200^TM^ 26/60-pg (GE Healthcare, Little Chalfont, UK), eluted in NaPB 7.0 containing 150 mM NaCl (PBS 7.0). For the final purification step, the active gel-filtered fractions were pooled, dialyzed against NaPB 7.5, filtered on a 0.22 µm filter and then applied to a Source^TM^ 15Q column, using the same conditions described above. Fractions from all steps were analyzed for ureolytic activity and submitted to 12% SDS-PAGE (Supplementary Figure S2). The active fractions were pooled and designated as purified PMU.

### 3.4. Protein Determination

The protein contents were determined by absorbance at 280 nm or by the Bradford method [44].

### 3.5. SDS-PAGE

Sodium dodecyl sulfate polyacrylamide gel electrophoresis (SDS-PAGE) was performed according to [45]. The material was diluted in a sample buffer, heated to 95 °C for 5 min and applied to 12 or 15% polyacrylamide gels. The gels were stained with colloidal Coomassie Brilliant Blue (Sigma-Aldrich, St. Louis, MO, USA).

### 3.6. Urease Assay 

Urease activity was determined in 96-well plates (Thermo Scientific, Waltham, MA, USA) in 100 µL (final volume) of PBS 7.4 containing 100 mM urea and the tested sample. After incubation (30 min, 37 °C), the color reaction was developed using the phenol-nitroprussiate method [46]. A standard curve was prepared with ammonium sulfate.

### 3.7. Platelet Aggregation 

Peripheral human blood of healthy volunteers was collected in 0.313% (*w*/*v*) sodium citrate. The blood samples were centrifuged at 400× *g* for 10 min at 25 °C to obtain a platelet-rich plasma (PRP). All procedures regarding blood collection and handling were conducted in strict accordance with the Brazilian legislation (Law no. 6.638/1979) and approved by the Institutional Ethics Committees (UFRGS 721.217; PUCRS 14/00414).

#### Platelet Aggregation by Turbidimetry

The method described in [31] was followed. Briefly, PRP aliquots (300 µL) were pre-incubated (2 min, 37 °C under stirring), and then a maximum of 30 µL of agonist was added. Aggregation of platelets was registered during 5 min in a Lumi-Aggregometer (Chrono-log Corporation, Havertown, PA, USA). The aggregation assays were performed with 11 or 63 nM of PMU. Adenosine diphosphate (ADP) at 20 µM was used as a positive control. Buffer alone was employed as the negative control.

### 3.8. Cell Cultures

BV-2 cells were kindly provided by Dr. Sandra Farsky, Universidade de São Paulo, Brazil. SH-SY5Y cells were kindly provided by Dr. Fabio Klamt, Universidade Federal do Rio Grande do Sul, Brazil. HEK293 cells were kindly provided by Dr. Douglas Sato, Pontificia Universidade Católica do Rio Grande do Sul, Brazil. The cell lines were maintained at 37 °C with 5% CO_2_. BV-2 cells were maintained in a RPMI-1640 medium (Sigma-Aldrich, St Louis, MO, USA) supplemented with 10% fetal bovine serum (FBS, Thermo Fisher Scientific, Grand Island, NY, USA) and 0.1% of pen-strep (Thermo Fisher Scientific, Grand Island, NY, USA). HEK293 and SH-SY5Y cells were maintained in DMEM (Thermo Fisher Scientific, Grand Island, NY, USA) supplemented with 10% FBS and 0.1% of pen-strep.

### 3.9. Cellular Viability

The method described in [35] was followed. Briefly, cells were treated for 24 h with NaPB 7.0 (control), 63, 126, or 252 nM PMU. The cultures’ supernatants were removed, and the cells were incubated with 3-(4,5-dimethylthiazol-2-yl)-2,5-diphenyl tetrazolium bromide (MTT, 5 mg/mL) (Sigma-Aldrich, St. Louis, MO, USA) for 4 h at 4 °C. The plates were centrifuged (2.000 rpm, 10 min, 4 °C) and the precipitates were resuspended with 100 µL 100% DMSO. The plates were read at 570 nm in a M2 spectrofluorometer (Molecular Devices, San Jose, CA, USA).

### 3.10. Intracellular Levels of Reactive Oxygen Species

The method described in [34] was followed. Cell cultures were incubated with the fluorophore probe 5-(and-6)-carboxy-2′,7′-difluorodihydrofluorescein diacetate (CM-DFFDA) (Thermo Scientific, Waltham, MA, USA) at 2 mM, for 30 min at 37 °C. After washing to remove the excess of fluorophore, the cells were incubated with NaPB 7.0 (control) or PMU, and 6 and 24 h afterwards the fluorescence (excitation 495 nm/emission 527 nm) was read in a M2 spectrofluorometer (Molecular Devices, San Jose, CA, USA). Readings of controls were considered as 1.0.

### 3.11. Determination of Intracellular Ca^2+^ Levels

The method of [47] was followed. Cells were incubated with the calcium fluorophore probe Fluo-4 (Thermo Scientific, Eugene, OR, USA) at 6 µg/µL, for 45 min at 37 °C. After washing, the cells were incubated with HEPES (145 mM NaCl, 5 mM KCl, 2 mM CaCl_2_, 1 mM MgCl_2_, 5 mM HEPES) pH 7.4 (control), 63, 126 or 252 nM PMU. The fluorescence (excitation 488 nm/emission 530 nm) was read at 0 h (right after treatment), 6 h and 24 h, in a M2 spectrofluorometer (Molecular Devices, San Jose, CA, USA).

### 3.12. Cytokines Determination

Cell cultures were incubated with NaPB 7.0 (control), 63, 126 or 252 nM PMU. After 6 h, the supernatants were collected and stored at −80 °C. To determine the levels of IL-1β and TNF-α, ELISA was performed using commercial kits (Invitrogen, California, USA, product code) according to the manufacturer’s instructions: human IL-1β (88–7261-88), human TNF-α (88–7346-88), murine IL-1β (88–7013-22) and murine TNF-α (88–7324-22).

### 3.13. Fluorescence Microscopy, Texas Red-Labeled PMU and Interaction with Cells

PMU (63 nM) or bovine serum albumin (BSA, 63 nM) was incubated with 0.5 mg/mL Texas Red (Sigma-Aldrich, St. Louis, MO, USA) for 1 h, at 4 °C, in the dark, with continuous stirring. The samples were then dialyzed against NaPB 7.0 to remove the free dye excess. Cell cultures were incubated with 63 nM Texas Red-labeled PMU or Texas Red-labeled BSA for 1 h at room temperature (r.t). After three washes with a buffer (30 min each, r.t), the cells were treated with 4% paraformaldehyde (10 min, r.t), incubated with ice-cold 100% methanol, and washed three times. After a blocking step (5% BSA, 0.3% Triton X-100) for 1 h at r.t., the cells were washed, and incubated with mouse anti-α-tubulin (Cell Signaling Technology, Danvers, MA, USA) diluted 1:1000 in PBS, 5% BSA, 0.1% Tween-20, for 1 h at r.t. Subsequently, the cells were washed three times and incubated with the secondary antibody (Alexa-Fluor 488-anti mouse, Molecular Probes Inc, Eugene, OR, USA) diluted 1:400 in PBS, 5% BSA, 0.1% Tween-20, washed three times, and stained with 300 nM DAPI (4′,6-diamidino-2-phenylindole) for 5 min. The cells were visualized under an inverted microscope Eclipse TE2000-S (Nikon, Tokyo, Japan) equipped with an ORCA-ER-1394 Camera (Hamamatsu Photonics K.K., Hamamatsu, Japan), with Phylum 4.2.0 (Improvision Inc., Lexington, MA, USA) as image acquisition software.

### 3.14. Analysis of PMU for Nuclear Localization Sequences 

The sequences of PMU subunits were retrieved from GenBank (access date: 21 April 2021) with the code M31834.1. The analyses for the prediction of the nuclear localization sequence were performed using cNLS Mapper (access date: 21 April 2021) [48]. The software defines a ranking from 1 to 10 where: 1, 2 are localized to the cytoplasm; 3, 4, 5 are localized to both the nucleus and the cytoplasm; 6, 7 are partially localized to the nucleus and 8, 9, 10 are exclusively localized to the nucleus [48].

### 3.15. Statistical Analysis

One-way ANOVA was used for comparisons and a * *p* < 0.05, ** *p* < 0.01 or *** *p* < 0.001 was considered statistically significant. Graphs and statistics tests were performed using GraphPad Prism 6 (San Diego, CA, USA). Results were expressed as mean ± standard error of the mean (SEM) and all the experiments were performed at least in triplicates.

## 4. Discussion

The well-recognized role of PMU as a virulence factor of *P. mirabilis* has been so far attributed exclusively to its enzyme activity. Here, our data revealed that PMU is a true moonlighting protein that carries several other biological properties unrelated to ammonia production that could potentially contribute to pathogenesis of urinary tract infection as well as to extra-urinary diseases associated to this bacterium.

Plant and bacterial ureases, regardless of their enzymatic activity, promote platelet activation by triggering an eicosanoid signaling cascade [26,31,36,38,39]. Particularly, the exocytosis-inducing effect of ureases that underlies platelet’s responses [26,31], and that correlates to their pro-inflammatory activity [27,28,32,33,34], could be relevant in the context of the diseases caused by *P. mirabilis*.

Here we showed the recombinant PMU-induced aggregation of human platelets in nanomolar concentrations (Figure 1A), which developed at a slower rate when compared to the faster response elicited by the platelet agonist ADP (at a 317-fold greater dose). Aggregation induced by HPU in rabbit [31] or in human platelets [32] also develops at a slower rate, suggesting that this may be a trend of platelets’ response to microbial ureases, contrasting with the much faster rate of canatoxin-induced response [39] (see Figure 1). A platelet-activating effect was reported for lipopolysaccharides extracted from *P. mirabilis* [49]. As the purity of the lipopolysaccharide preparations was not described, it is not possible to exclude the presence of low amounts of PMU in those samples. Thrombotic thrombocytopenic purpura (TPP) is a rare blood disorder caused by an acquired or congenital deficiency in ADAMTS13 activity which results in clotting in small blood vessels and inappropriate platelet aggregation, leading to thrombocytopenia. Bacterial infections, particularly urinary tract infections associated with urinary stones, have been postulated as potential causes of acquired TPP [50]. The role of platelet-activating ureases produced by urinary pathogens, such as *P. mirabilis,* in the pathogenesis of TPP has not been addressed so far.

The cytotoxic and pro-inflammatory activities of PMU were first examined in human embryonic kidney HEK293 cells. The dose range chosen for PMU considered that the non-enzymatic biological properties determined for other ureases in several different in vitro models occurred in the 10–300 nM range, ranged in periods spanning a few seconds (i.e., platelet aggregation) to up to a couple of days. While not affecting cell viability (as indicated by the mitochondrial function assay, Figure 3A) at the highest dose after 24 h exposure, HEK293 cells responded to PMU in a dose- and time-dependent way. The effects of PMU on HEK293 cells were independent of its enzymatic activity as no increase in the ammonia levels in the culture medium was observed (Figure 2). Furthermore, our data show that HEK293 cells acquired a pro-inflammatory phenotype in the presence of PMU, producing reactive oxygen species and secreting IL-1β, and TNF-α (Figure 3B–D). The pro-inflammatory effect of PMU occurred in concentrations as low as 126–252 nM PMU and increased with the incubation time. Thus, besides the toxicity caused by the generated ammonia to the urinary tract tissues, PMU may aggravate the tissue damage through other non-enzymatic effects.

The contribution of the platelet- and neutrophil-activating properties of ureases to inflammation was previously demonstrated for canatoxin [27,28,38,39] and HPU [31,32,33,34]. Both ureases were shown to induce eicosanoid-dependent paw edema in rodents, with an intense infiltration of neutrophils. The activation of human neutrophils by HPU does not require the enzyme’s activity, leading to an increased lifespan and extracellular production of oxygen reactive species by the leukocytes [34]. Human platelets activated by HPU presented increased processing of pre-mRNA of IL-1β and CD14, indicating conversion to a pro-inflammatory phenotype [32]. Human microvasculature endothelial cells also showed inhibition of apoptosis and augmented production of ROS, nitric oxide, and IL-1β upon treatment with HPU at 10 nM [33]. Noteworthy, inflammation and tissue damage associated with *P. mirabilis* infections are typically characterized by recruitment of inflammatory monocytes and increased IL-1β production by the NLRP3 inflammasome [9]. Thus, although no information is available on the concentrations of PMU in the urine of patients with *P. mirabilis* infection, its eventual contribution in worsening the inflammation of the urinary tract caused by this bacterium should not be overlooked.

The extra-urinary pathologies associated with *P. mirabilis* infections include some neurological conditions. For instance, *P. mirabilis* is the cause of ~4% of neonatal meningitis due to Gram-negative bacteria [10] and of ~7% of adult meningitis following neurosurgery [12,51]. A possible association of *P. mirabilis* with Parkinson’s disease (PD) has been reported. Gut microbiota studies in PD patients showed a specific increase in bacteria of the Enterobacteriaceae family, to which *P. mirabilis* belongs [16,52]. Moreover, Choi and co-workers reported in 2017 that oral administration of *P. mirabilis* to mice resulted in motor deficits, selective death of dopaminergic neurons, and increased contents of fibrillar α-synuclein in the colon and the brain, a hallmark of this disease [15]. Additionally, Pan-Montojo and colleagues demonstrated that PD pathology possibly starts in the gut, with the release of α-synuclein by enteric neurons and the migration of the toxin through the vagus nerve to the brain [53]. In this context, a potential contribution of neurotoxic effects of PMU to these neurological diseases deserves investigation.

Here, we used human neuroblastoma SH-SY5Y and murine microglia BV-2 as models of CNS cells. PMU did not affect their viability in any of the tested doses and time schedules. Although the mitochondrial function (indicative of viability) seemed preserved (Figure 4A), PMU induced neuroblastoma cells to produce intracellular reactive oxygen species in all evaluated doses and time-points (Figure 4C). On the other hand, BV-2 microglial cells did not produce reactive oxygen species under the same test conditions (Figure 4D). It is worth noting that, although neither lineage was affected in the MTT assay, the neuroblastoma cells had a slightly increased mitochondrial activity. This may correlate with the increase in ROS production seen only for the SH-SY5Y cells.

Microglial BV-2 cells, but not neuroblastoma SH-SY5Y cells, released the pro-inflammatory cytokines IL-1β and TNF-α (Figure 4E–H). Microglia are considered the immune cells of the CNS, so a PMU-induced increase in the release of pro-inflammatory cytokines in BV-2 cells was expected. This result goes in hand with the previously described induction of cytokine release by HPU-stimulated immune cells [54,55,56]. The activation of NLRP3 and the release of IL-1β by the B subunit of HPU was reported in dendritic cells [57]. Different kinetics of cytokine production and the time point (6 h) chosen for our study could be a reason why no IL-1β or TNF-α was detected in the supernatant of SH-SY5Y cells treated with PMU. This neuroblastoma cell line is known to secrete TNF- α and IL-1β, regardless of differentiation [58,59,60].

The levels of intracellular calcium ions were determined for the three types of cells, HEK293, SH-SY5Y, and BV-2, to reflect their activation status upon exposure to PMU. As seen in Figure 5, only neuroblastoma cells had an increase in the intracellular levels of calcium, probably reflecting an influx of external ions. We have previously reported in platelets stimulated by canatoxin [61] or by *H. pylori* urease [31], an increased influx of external calcium inhibitable by D-methoxyverapamil. The reason why only SH-SY5Y cells responded to PMU with an increased calcium content remains elusive. Reports are showing that undifferentiated neuroblastoma cells have L-type and T-type voltage-gated calcium channels [62,63]. Although HEK293 and BV-2 cells do not elicit action potential, they have calcium channels that could lead to increased intracellular ion levels. For instance, store-operated calcium channels were reported in HEK293 cells [64,65,66]. Calcium levels regulate many functions of microglial cells, and it is known that murine BV-2 cells display purinergic ligand-gated calcium channels [67] as well as transient receptor potential calcium channels [68].

The subcellular localization of PMU in the different cells was very similar, as revealed by fluorescence microscopy (Figure 6A–C). After 1 h of exposure, PMU was found inside all three types of cells, distributed in the cytoplasm and the nucleus. We previously reported that cultures of K562 (human erythroleukemia), SP2 (murine plasmacytoma), and EL4 (murine lymphoma) cells internalized canatoxin (100–500 nM) upon 30 min of exposure to the protein. Immunofluorescence microscopy showed binding of canatoxin to the cell membrane, followed by patching, capping, and internalization [69]. Immunofluorescence studies also showed the internalization of *H. pylori* urease (100 nM) by AGS gastric epithelial cells after 30 min [35].

It has been previously reported that the A subunit of HPU contains a monopartite nuclear localization signal (sequence 21KKRKEK26) and that HPU was found in the nuclei of COS-7 and AGS cells, causing alterations of the cellular morphology [41,70]. Extending these previous studies, here we described two conserved bipartite NLS in HPU, one of which contains the reported sequence 21KKRKEK26. The two NLS sequences found in PMU and HPU are evolutionarily conserved, showing 65.6 and 55.9% identity of their sequences, respectively (Figure 7 and Supplementary Figure S3). Thus, besides promoting inflammation, nuclear targeting of PMU could potentially lead to effects on gene expression, thereby affecting the normal physiology of cells in the urinary tract or other cell types in the case of extra-urinary pathologies caused by *P. mirabilis*.

## 5. Conclusions

We believe that the relevance of PMU as a virulence factor has been so far underappreciated and that this protein could probably be involved in many other features of *P. mirabilis* pathogenesis than merely providing nitrogen and shelter (by forming urinary stones) for the bacteria. In vivo approaches such as experimental infection with *P. mirabilis* in animals immunized against PMU, infection with PMU-silenced bacteria, and/or in vivo studies with the purified protein could help deepen our understanding of the role of PMU as a virulence factor.

## Data Availability

Data is contained within the article or supplementary material.

## Supplementary Materials

The following are available online at https://www.mdpi.com/article/10.3390/ijms22137205/s1.
