# Supplementary material for: Proteus mirabilis Urease: Unsuspected Non-Enzymatic Properties Relevant to Pathogenicity
Source: Int J Mol Sci. 2021 Jul 4;22(13):7205. doi: 10.3390/ijms22137205 (PMC8268090; doi:10.3390/ijms22137205)
Supplement: Supplementary file 1 [file ijms-22-07205-s001.zip › ijms-1274755-supplementary.pdf]

International Journal of *Molecular Sciences*

MDPI

Supplementary Material

Proteus mirabilis urease: Unsuspected non-enzymatic properties relevant to pathogenicity

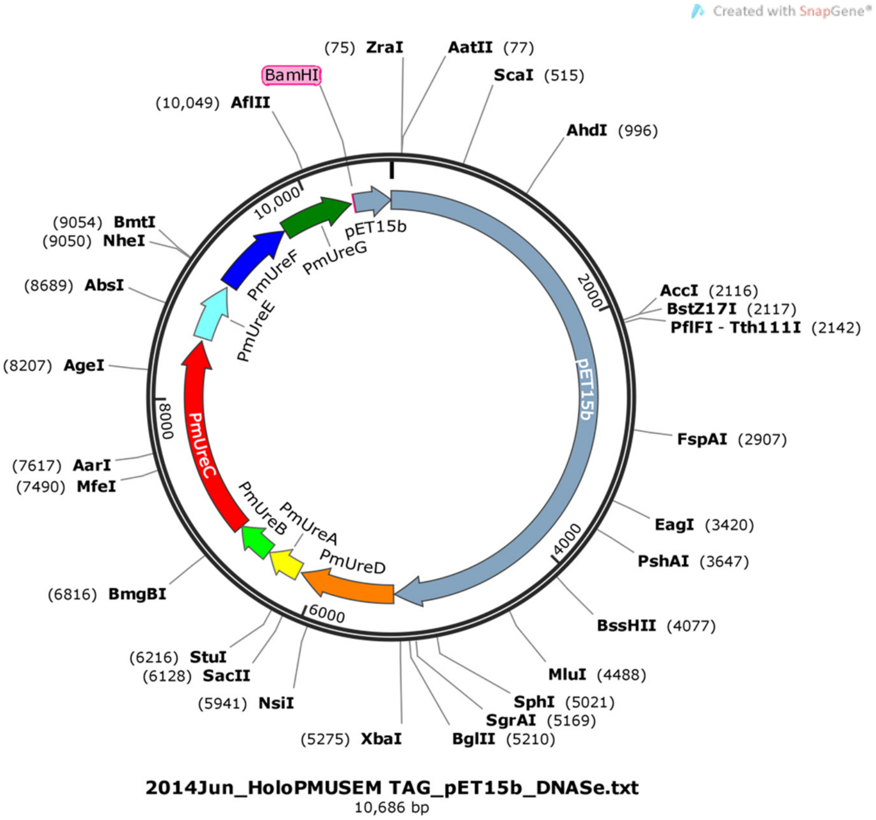

Supplementary Figure S1. Restriction Map of the D*ureR* holoPMU::pET15b plasmid

The modified operon f D*ureR* holoPMU contains seven genes in tandem *ure*D-*ure*ABC-*ure*EFG: three structural genes (*ure*A, *ure*B and *ure*C) and four genes encoding accessory and regulatory proteins (*ure*D, *ure*E, *ure*F, and *ure*G), thus encoding a fully active *Proteus mirabilis* urease (PMU) urease. The modified operon was inserted into a pET15b plasmid

between XbaI and BamHI restriction sites. The figure was prepared with the Snapgene software (from Insight Science; available at www.snapgene.com).

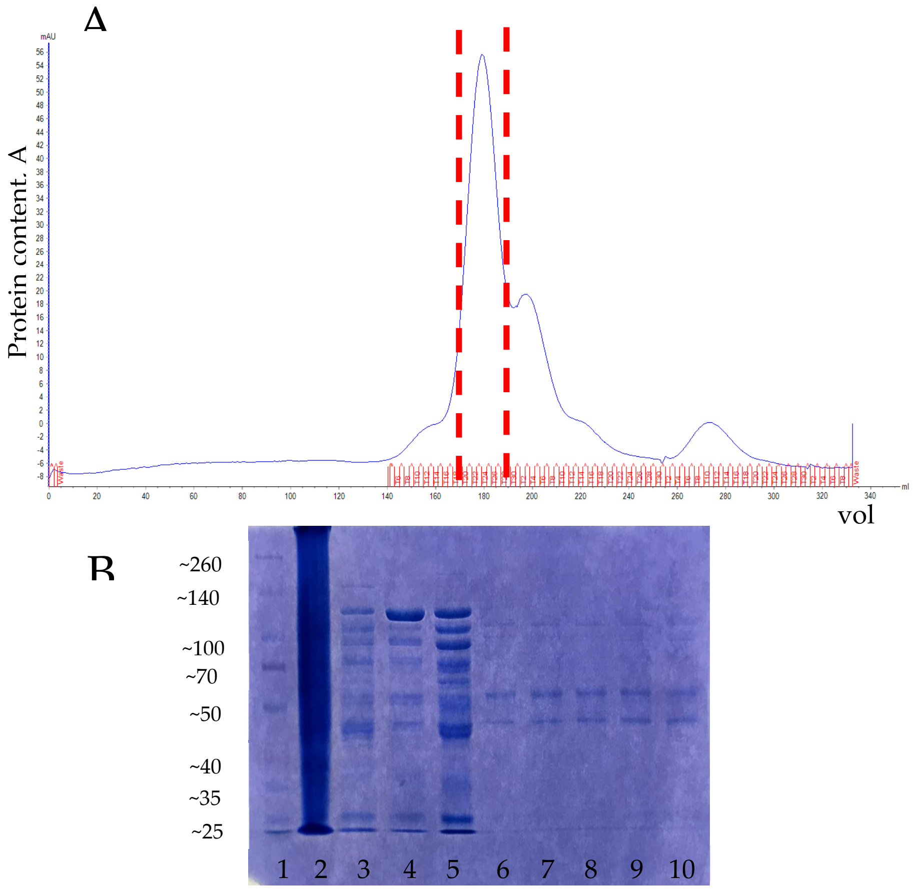

Supplementary Figure S2. Purification of recombinant *P. mirabilis*

Purification PMU consisted of a sequence of ion exchange and molecular size exclusion chromatographies. Panel A shows the chromatographic patterns obtained in the Superdex 200™ 26/60 column. The dashed lines indicate fractions with ureolytic activity. Panel B shows a SDS-PAGE 12 % of samples during the purification protocol (1- Molecular mass markers, 2- crude extract, 3- active pool after Q-Sepharose column, 4 and 5- active pool from the first Source 15Q column, 6,7,8,9 and 10 are fractions from the Superdex S200 column). The protein band with Mr ~60kDa in lanes 6-10 corresponds to subunit C of *P. mirabilis* urease.

```
HPU    1 MKLTPKELDKLMLHYAGELAKKRKEKGIKLNYVEAVALISAHIMEEARAG   50
         |:|||:|.|||:|..||.:|::|..||:||||.|.|||||..|||.||.|
PMU    1 MELTPREKDKLLLFTAGLVAERRLAKGLKLNYPERVALISCAIMEGAREG   50

HPU   51 KKTAAELMQEGRTLLKPDDVMDGVASMIHEVGIEAMFPDGTKLVTVHTPI  100
          ||.|:||.||||:|..:.||:||..||.:|.:|..||||||||::|:||
PMU   51 -KTVAQLMSEGRTVLTAEQVMEGVPEMIKDVQVECTFPDGTKLVSIHSPI   99

HPU  101 EANGKLVPGELFLKNE--DITINEGKKAVSVKVKNVGDRPVQIGSHFHFF  148
             .::|||:.:...  ||.:|.|::..:::|.|.||||||:|||:||:
PMU  100 ----VMIPGEIRVNAALGDIELNAGRETKTIQVANHGDRPVQVGSHYHFY  145
              ↑1

HPU  149 EVNRCLDFDREKTFGKRLDIAAGTAVRFEPGEEKSV-ELIDIGGNRRIFG  197
         |||..|.|.|::|.|.||:|.||.|||||||:.::| ||:...|.|.|:
PMU  146 EVNEALRFARKETLGFRLNIPAGMAVRFEPGQSRTVDELVAFAGKREIY-  194
                                                  ↓2
HPU  198 FNALVDRQADNESKKIALHRAKERGFHGAKSDDNYVKTIKEMKKISRKEY  247
                                 ||||         .:..||.|||:.|
PMU  195 ------------------------GFHG---------KVMGMKTISRQAY  211
                                                ↑3

HPU  248 VSMYGPTTGDKVRLGDTDLIAEVEHDYTIYGEELKFGGGKTLREGMSQSN  297
         ..|:||||||::||.||:|..|:|.|:|.||||:||||||.:|:||.||.
PMU  212 ADMFGPTTGDRLRLADTELFLEIEKDFTTYGEEVKFGGGKVIRDGMGQSQ  261

HPU  298 NPSKEELDLIITNALIVDYTGIYKADIGIKDGKIAGIGKGGNKDMQDGVK  347
         ..|.|.:|::||||:|:||.||.|||||||||:|.||||.||.|:|..|
PMU  262 VVSAECVDVLITNAIILDYWGIVKADIGIKDGRIVGIGKAGNPDVQPNV-  310

HPU  348 NNLSVGPATEALAGEGLIVTAGGIDTHIHFISPQQIPTAFASGVTTMIGG  397
          ::.:||.||.:||||.||||||||||||||.|||......|||||.|||
PMU  311 -DIVIGPGTEVVAGEGKIVTAGGIDTHIHFICPQQAQEGLVSGVTTFIGG  359

HPU  398 GTGPADGTNATTITPGRRNLKWMLRAAEEYSMNLGFLAKGNASNDASLAD  447
         ||||..||||||:|||..|:..||.|.:|..:|:|...||..|...::.:
PMU  360 GTGPVAGTNATTVTPGIWNMYRMLEAVDELPINVGLFGKGCVSQPEAIRE  409

HPU  448 QIEAGAIGFKIHEDWGTTPSAINHALDVADKYDVQVAIHTDTLNEAGCVE  497
         ||.|||||.|||||||.||.||::.|:|||:.|||||||:|||||.|..|
PMU  410 QITAGAIGLKIHEDWGATPMAIHNCLNVADEMDVQVAIHSDTLNEGGFYE  459

HPU  498 DTMAAIAGRTMHTFHTEGAGGGHAPDIIKVAGEHNILPASTNPTIPFTVN  547
         :|:.|||||.:|.|||||||||||||:||..||.||||||||||:|:|:|
PMU  460 ETVKAIAGRVIHVFHTEGAGGGHAPDVIKSVGEPNILPASTNPTMPYTIN  509

HPU  548 TEAEHMDMLMVCHHLDKSIKEDVQFADSRIRPQTIAAEDTLHDMGAFSIT  597
         |..||:||||||||||.||.|||.||:||||.:||||||.||||||.|:.
PMU  510 TVDEHLDMLMVCHHLDPSIPEDVAFAESRIRRETIAAEDILHDMGAISVM  559

HPU  598 SSDSQAMGRVGEVITRTWQTADKNKKEFGRLKEEKGDNDNFRIKRYLSKY  647
         ||||||||||||||.||||.|.|.|.:.|.|..:..||||.|||||::||
PMU  560 SSDSQAMGRVGEVILRTWQCAHKMKLQRGTLAGDSADNDNNRIKRYIAKY  609

HPU  648 TINPAIAHGISEYVGSVEVGKVADLVLWSPAFFGVKPNMIIKGGFIALSQ  697
         |||||:||||:..|||:|.||:||:|||.||||||||.:|||||.:..:.
PMU  610 TINPALAHGIAHTVGSIEKGKLADIVLWDPAFFGVKPALIIKGGMVRYAP  659

HPU  698 MGDANASIPTPQPVYYREMFAHHGKAKYDANITFVSQAAYDKGIKEELGL  747
         |||.||:|||||||:||.|:|..|||||..::.|:|:|..:.|:.|:|||
PMU  660 MGDINAAIPTPQPVHYRPMYACLGKAKYQTSMIFMSKAGIEAGVPEKLGL  709

HPU  748 ERQVLPVKNCRNVTKKDMQFNNTTAHIEVNPETYHVFVDGKEVTSKPANK  797
         :..:..|:.||::||..|..||...|||::|:||.|..||..:..:||.:
PMU  710 KSLIGRVEGCRHITKASMIHNNYVPHIELDPQTYIVKADGVPLVCEPATE  759

HPU  798 VSLAQLFSIF    807
         :.:||.:.:|
PMU  760 LPMAQRYFLF    769
```

Supplementary Figure S3. Alignment of amino acid sequences of *Proteus mirabilis* and *Helicobacter pylori* ureases

Protein sequences for bacterial urease subunits were collected from NCBI - Protein database (https://www.ncbi.nlm.nih.gov/protein/) and aligned using the EMBOSS Needle algorithm [1]. *Proteus mirabilis* urease (GenBank: M31834.1), *Helicobacter pylori* urease (PDB 19EZ). Conserved amino acid positions are marked (|) for identity,(:) for strong similarity and (.) for similarity. Sequences targeting intracellular compartments are shown in bold underlined letters. The NLS in the N-terminal domain of HPU (positions 21-56) and PMU (positions 22-54) were predicted by cNPS mapper with scores of 4.9 and 4.7, respectively. The sequence 21KKRKEK26 in HPU was previously identified as a NSL [2]. The NLS in the C-terminal domain of HPU (positions 613-646) and PMU (positions 575-608) were predicted by cNPS mapper with scores of 4.4 and 5.6, respectively. Arrows 1 and 2 indicate the N-terminal Met residues of PMU's subunit B and C, respectively, and arrow 3 indicates the N-terminal Met residue of HPU's subunit B.

Reference

1. Needleman, S.B.; Wunsch, C.D. A general method applicable to the search for similarities in the amino acid sequence of two proteins. *J. Mol. Biol.* **1970**, *48*, 443–453, doi:10.1016/0022-2836(70)90057-4.
2. Suarez, I.; Bodega, G.; Fernandez, B. Glutamine synthetase in brain: Effect of ammonia. *Neurochem. Int.* **2002**, *41*, 123–142, doi:10.1016/s0197-0186(02)00033-5.
